# Preparation and Evaluation of A Novel Liposomal Nano-Formulation in Metastatic Cancer Treatment Studies

**DOI:** 10.22074/cellj.2019.6008

**Published:** 2019-02-25

**Authors:** Fatemeh Barzegari Firouzabadi, Shahrbanoo Oryan, Mohammad Hasan Sheikhha, Seyed Mehdi Kalantar, Ameneh Javed

**Affiliations:** 1 Department of Animal Biology, Faculty of Biological Science, Kharazmi University, Tehran, Iran; 2 Departeman of Biology, College of Science, Payame Noor University, Yazd, Iran; 3 Reproductive and Genetic Unit, Research and Clinical Center for Infertility, Shahid Sadoughi University of Medical Sciences, Yazd, Iran; 4 Department of Biology, Faculty of Science, Science and Art University, Yazd, Iran

**Keywords:** Cell Survival, Liposome, microRNA, Osteosarcoma

## Abstract

**Objective:**

Today, in clinical trials, we suffer from the lack of effective methods with minimal side effects to deliver medication. Thus, efforts to identify better conditions for delivery of biomedical drugs seem necessary. The purpose of this study was to design a new liposomal formula for transportation of microRNA in osteosarcoma.

**Materials and Methods:**

In this experimental study, several liposomal formulations were synthesized. Physical and chemical parameters, including size, zeta potential, polydispersity index, long-term stability of the liposomal-microRNA complex and the amount of *miR-143* loading in liposome based nano-vesicles were optimized using different techniques. Similarly, the effect of free and encapsulated microRNA toxicity were investigated and compared in a human bone osteosarcoma cell line, named SaOs-2.

**Results:**

In this study, we could produce a novel and optimized formulation of cationic PEGylated liposomal microRNA for gene delivery. The present synthesized microRNA lipoplex system was non-agglomerated. The system remained stable after four months and *miR-143* leakage was not observed by performing gel electrophoresis. The microRNA lipoplex could enhance conduction of the loaded *miR-143*, and it also showed good biocompatibility to the healthy cells.

**Conclusion:**

The PEGylated microRNA lipoplex system had a high potential for the systematic migration of *miR-143* and it could improve intracellular stability of the released microRNA.

## Introduction

Cancer is a disease in which the cells begin to grow and divide due to the various causes and continuously produce abnormal cells (1). Despite the endless efforts of scientists to treat cancer, there is no therapeutic system yet for the successful treatment of cancer, thus making this disease one of the leading causes of mortality worldwide. Although many molecular factors of this disease have been discovered, it is still very difficult to detect many indirect and direct factors in the development of cancer, and in many cases, providing physicians unable to cure this disease (2, 3).

Several systemic and topical malformations related to bone have turned it into a crucial subject to study. From higher to lower degree, bones could contribute to different diseases, caused by genetic or environmental factors. Bone cancer is a type of malignancy spreading to bone from another cancerous tissue (4, 5). Compared to the metastatic bone cancer, more commonly observed in adolescent, osteosarcoma is a malignant tumor, with childhood and adulthood onset. So that 75% of the patients are less than 20 years old (6, 7). Since osteosarcoma is involved in the community of children and adult population, it is important to study different treatment approaches for this disease.

Gene therapy is a biological method for treatment of disease through repairing and eliminating gene defects. Gene therapy strives to treat abnormal cells by inserting oligo-or poly-nucleotide fragments. In gene therapy, selecting an appropriate carrier with high transfection efficiency and minimal toxicity is very important. In past, viral carriers were used for gene transfer. Although, viral vectors have high efficiency for gene transfer, potential of oncogenesis ability, high cost and limitations in transported DNA size are some disadvantages of their application. At present, researchers are paying more attention to non-viral polymeric carriers, including liposomes. These carriers have higher biocompatibility than viral types, while they have low level of gene expression (8, 9).

Liposomes are bilayer polymeric vesicles that can be used for loading different biological molecules, including microRNAs. In case of utilizing cationic lipids, the resultant particle would have a positive charge net and the negative charge of the nucleic acids is compensated facilitating cellular absorption. This strategy is used with high success rate probability for microRNAs, as therapeutic agents for cancer treatment (10). microRNAs include short non-coding RNAs (about 21-25 nucleotides) that are commonly used as inhibitor of target gene mRNAs. This procedure is mainly performed by influencing the stability and translation of mRNAs at post-transcriptional levels. During the past few years, study of microRNAs in human cancers has revealed that many of them act as tumor suppressors (11, 12). *miR-143* is a well-known tumor suppressor that neutralizes tumor progression by regulating a number of oncogenes. It has been shown that *miR-143* prevents tumorigenicity by targeting the *N-RAS* gene in glandular cancer (13) and *COX2* gene in intestinal cancer (14). In the present study, *miR-143* (cancer inhibitor) loaded liposomal system (microRNA lipoplex) was designed and targeted against human bone sarcoma SaOs-2 cell line, in order to obtain a targeted drug delivery system and to reduce the harmful effects of chemotherapeutics.

## Materials and Methods

### Cell line 

Human bone sarcoma SaOs-2 cell line was obtained from the National Cell Bank of Iran (NCBI), Pasteur Institute, Tehran, Iran. Human primary osteoblast (Hum-63 cell line), as short-term culture, were kindly provided by Shahid Sadoughi University of Medical Sciences, Yazd, Iran. Cells were incubated (Matter, Germany) at 37°C and 5% CO_2_ in the Dulbecco’s Modified Eagle’s medium (DMEM, Gibco Invitrogen, Germany), containing fetal bovine serum (FBS, Sigma, USA), augmented with penicillin and streptomycin (both from sigma, USA). After three successive passages, the cells were treated with microRNA lipoplex system. In this research, ethical considerations are approved based on the International Campus of Yazd University of Medical Sciences, Yazd, Iran. 

### Chemicals

Cholesterol,1,2-dioleoyl-3-trimethylammoniumpropane (DOTAP), 4% paraffinic acid solution and fluorescent label (Dil) were respectively purchased from Sigma-Aldrich and Avanti Polar Lipids (both from USA). Polyethylene glycol (PEG) and dipalmitoyl phosphatidylcholine (DPPC) were purchased from Lipoid (GmbH, Germany). The 4, 6 diamidino-2-phenylindole (DAPI) was purchased from Thermo Fisher, USA. All other chemicals and solvents, used in this study, were of the highest purity and analytical grade. microRNA mimic, hsa-*miR-143* and CY-5 *miR-143* (microRNA conjugate with red fluorescent dye) was purchased from Sigma- Aldrich, USA. 

#### Synthesis of uniform nano-liposomal formulation

##### Preparation of thin lipid film 

In this experimental study, lipid phase consisting of PEG, cholesterol (Chol), DOTAP, DPPC, DSPE-mPEG (2000) and different DOTAP concentrations (0, 30, 40 and 50%), dissolved in chloroform. DOTAP is a cationic phospholipid used in this liposome formulation to generate positive bands in the system. Then, the organic phase of solution was removed using a rotary evaporator and a thin lipid film was formed on the balloon wall. To ensure complete solvent removal, the thin lipid film was aerated for several minutes with nitrogen gas and placed at 4°C for 24 hours.

#### Hydration of lipid film and reducing the size of liposomal products

The liquid phase for hydration of the thin film was composed of phosphate buffered saline (PBS) with pH=7.4. Immediately after addition of PBS, a milky fluid was formed, which was identical to the multilamellar vesicles (MLV) liposomes. To reduce the size of MLV and small unilamellar vesicles (SUV), the resultant samples were sonicated. To prevent unwanted rise in temperature during sonication, the balloons containing nano-liposomes were placed in an ice container under 60% amplitude for 20 minutes (7 seconds ON and 10 seconds OFF). 

#### Determination of physico-chemical characteristics of nano-liposomes

##### Determination of particle size distribution 

To measure size of the nano-liposomal specimens and liposome-gene complex, the samples were first diluted twice with distilled water (DW). To measure liposomal size, final concentration of the samples was 0.225 mg/ml. Thus at this concentration, liposomal size was not affected by their concentration. In contrast, an error was observed at higher or lower concentrations, due to inaccurate calculation. For that, dilution was performed before size analysis. Hydrodynamic diameter and polydispersity index (PDI) of the specimens was determined by Dynamic Light Scattering (DLS, Brookhaven Corp, USA) at room temperature. All measurements were repeated four times.

#### Zeta potential measurements 

The surface charge and Zeta potential of nanoliposomes was measured using a Zeta Sizer (Brookhaven Instruments, USA) at 25°C. To determine surface charge, 1500 µl samples were used with 0.1 µg/ml concentration. Each parameter was measured thrice. 

#### Morphological evaluations 

Shape and surface morphology of the synthesized nano-liposome system were evaluated by Field Emission Scanning Electron Microscopy (FESEM, KYKYEM3200- 30KV, China).

#### Formulation of microRNA containing lipoplex 

For microRNA loading into the cationic nanoliposomes, different ratios of SUV liposome to microRNA were incubated at ambient temperature for 30 minutes. To increase stability time and uniformity of the products, cationic nano-liposomes were filtered five times with the extruder before incubation.

### Optimization of microRNA loading into nanoliposomes with agarose gel electrophoresis

To determine optimal dosage of loading microRNA into nano-liposomes, various microRNA and liposomal ratios were casted onto the agarose gel (2%) electrophoresis with ethidium bromide (30 minutes electrophoresis at 80 volts). Briefly, 5 µl of a suspension containing liposome-*miR-143* complex, was combined with 1 µl of loading buffer (Biolabs, UK). Different concentrations of microRNA and liposome were analyzed to determine the most appropriate liposomal concentration for microRNA loading. After completion of agarose electrophoresis, the gel was transferred to gel documentation system (UVP, UK) and the results were analyzed.

### Physical stability of lipoplex 

#### Leakage stability

Ability of the lipoplex system to preserve *miR-143* was monitored for 4 months at 4°C, and microRNA leakage from lipoplex was evaluated by electrophoresis.

### Stability in mouse serum 

To study stability of the designed nano-systems in conditioned *in vitro* environment, the lipoplex was kept in mouse serum (at 37°C) for different time periods (one, two and four hours), then put into 6-well cell culture plates containing SaOs-2 cell line, and placed in the humidified 37°C incubator under 5% CO_2_ for 1 hour. The cells were then washed 3 times with PBS and fixed with 4% paraformaldehyde solution (Thermo Fisher Scientific, USA) for 15 minutes. Fluorescence microscope (Olympus, Japan) was employed to observe the samples.

### Investigation of cytotoxicity after nano-liposome administration 

Hum-63 cells were cultured in 96-well microplates, containing DMEM, modified with 10% FBS, 1% penicillin/streptomycin (Gibco Invitrogen, USA) at 37°C and 5% CO_2_. Repeatability, accuracy and sensitivity of the tests were high. MTT assay was used to determine cytotoxicity after 48 and 72 hours of Hum-63 cell treatment with different concentrations of empty liposomes. Plates were placed in incubator for 24, 48 and 72 hours. After each incubation period, the cells were washed twice with PBS and 20 µl of 5 mg/ml MTT (diluted with PBS) and they were added to each well and the plates were incubated for four hours to allow the formazan crystal formation. After completion of four hours incubation, the internal solution of each well was completely removed and 200 µl dimethyl sulfoxide (DMSO) was added to dissolve the crystals. The resultant samples were studied with microplate ELISA reader (Biotek Instruments Inc, USA), and the cell viability percentage was calculated using the following formula: 

$$\mathrm{\% Viable cells}=\frac{\mathrm{Mean optical absorption in the test group}-\mathrm{Average light absorption in culture medium}}{\mathrm{Mean optical absorption in the control group}-\mathrm{Average light absorption in culture medium}}\times 100$$

### Comparative study of the lipoplex system effect 

SaOs-2 cells were seeded in 96-well plates for 24 hours to adhere to the plate bottom. The medium was next replaced with fresh culture medium (in control), or treated with a lipoplex complex, free microRNA and empty liposomes. After 72 hours MTT analysis was performed, as stated above and the resultant absorption was recorded at 570 and 630 nm wavelengths.

### Investigation of microRNA delivery into bone cancer cells through lipoplex system

Bone cancer SaOs-2 cell line was used to study lipoplex transfection. Firstly, sterile glass lamella was placed inside the wells of a 6-well plate, and the cells were counted and added at the concentration of 5×10^5^ cells/well. After 24 hours, the culture medium was drained and the cells were exposed to liposomal CY-5 microRNA, diluted with culture medium. The cells were incubated for 3 hours at 37°C and they were then washed thrice with cold PBS and fixed with paraformaldehyde solution. DAPI solution (125 µg/ml) was used for 15 minutes to stain the cell nuclei. The efficacy of microRNA cell transfection was evaluated using a fluorescence microscope (Olympus, Japan). 

### Statistical analysis

For statistical analysis of the data, SPSS for Windows was used and the paired t test was used to compare different groups, where P<0.05 was considered statistically significant.

## Results

### Zeta potential and particle size analysis

**Fig.1:**
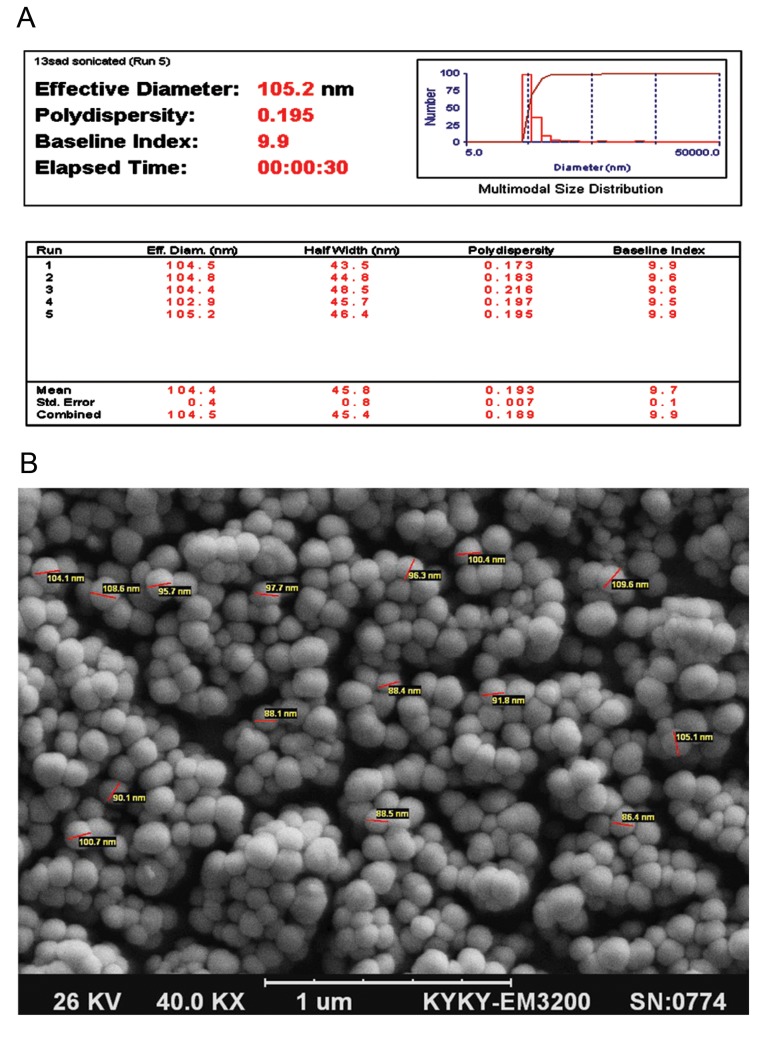
Particle size and SEM microscope analysis. A. DLS analysis of optimal formula and B. SEM micrograph of the newly formulated nano-liposomes. SEM; Scanning electron microscope and DLS; Dynamic light scattering.

Liposomes were prepared with DPPC, cholesterol, DOTAP and DSPE-mPEG. Table 1 shows characteristics of the various formulations, synthesized for carrying microRNA. In addition, Figure 1A shows DLS analysis of optimal formula. According to the results, with increasing DOTAP concentration, surface charge of liposomes is also increased. DOTAP molecule with a positive agent group produced a positive charge in the liposomal structure. In all cases, dispersion index was less than 0.3 which indicates no agglomeration in liposomal particles, and zeta potential was positive, while it was reduced after incubation with microRNA (15-17). PEGylation reduced PDI of liposomes, due to the increase in repulsive force of particles with a positive charge. Additionally, spatial blocking prevented accumulation of the particles (18).

Electron micrograph of nano-liposomes is shown in Figure 1B. SEM micrograph confirmed the size and spherical shape of the newly nano-formulated system. 

**Table 1:** Characterization of liposomal formulations


Code	DPPC (g)	Chol (g)	DOTAP (g)	PEG (g)	Zeta potential (mv)	Size (nm)	PDI	Total volume (ml)

F1	0.0102	0.0023	0	0	-24.33 ± 0.83	148.4 ± 2.4	0.297 ± 0.01	1
F2	0	0.0038	0.0071	0	+38.23 ± 0.33	125.22 ± 2.3	0.230 ± 0.024	1
F3	0.0061	0.0013	0.0061	0	+34.21 ± 0.23	130 ± 1.3	0.202 ± 0.02	1
F4	0.0071	0.0016	0.0046	0	+21.87 ± 1.43	114.67 ± 1.60	0.128 ± 0.01	1
F5	0.0066	0.0015	0.0046	0.0028	+29.32 ± 1.02	119.52 ± 0.8	0.107 ± 0.01	1
F6	0.0068	0.0015	0.0046	0.0016	+27.24 ± 0.21	105.23 ± 0.36	0.109 ± 0.03	1
F6 with microRNA	0.0068	0.0015	0.0046	0.0016	+13.61 ± 0.33	137.45 ± 0.51	0.110 ± 0.02	1


DPPC; 1,2- Dipalmitoyl-sn-glycero-3 phosphocholine, DOTAP; 1,2-dioleoyl-3-trimethylammonium-propane, PEG; DistearoylPhosphoethanolamine (PE 18:0/ 18:0-PEG2000, DSPE-mPEG 2000), and PDI; Dispersion index.

### Investigation of cell survival after liposomal treatment 

**Fig.2:**
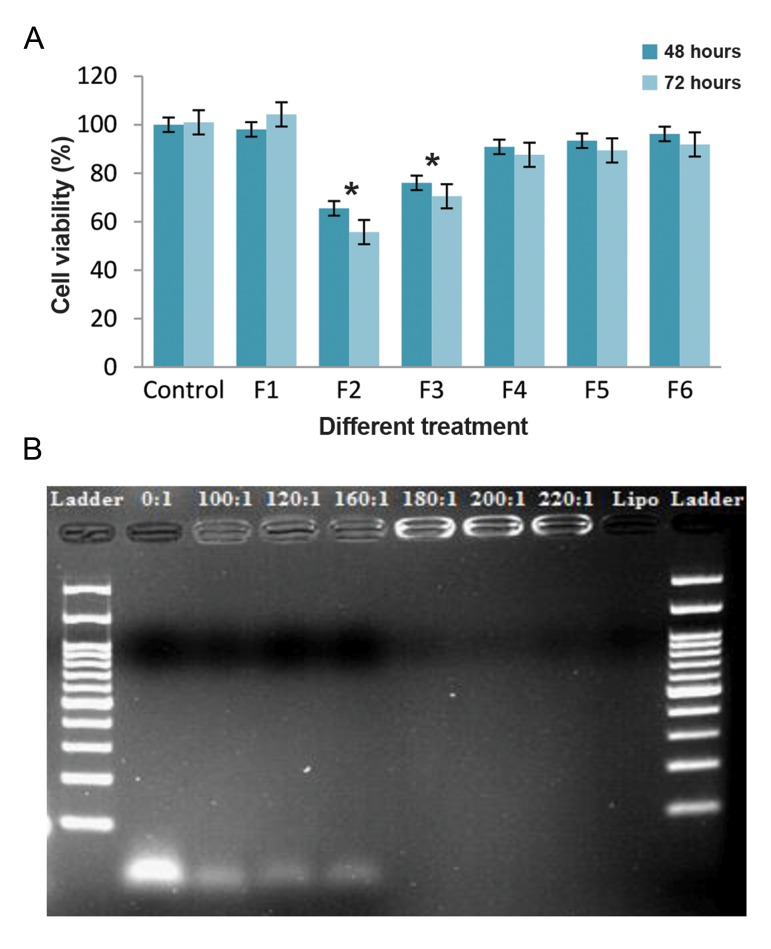
Investigation of cell survival after liposomal treatment and optimization of microRNA loaded into nano-liposomes. A. Comparison of the toxicity impact of various empty liposome formulations after 48 and 72 hours in hum-63 cells and B. Optimization of loading microRNA into nano-liposomes with agarose gel electrophoresis to determine the maximum effective concentration of liposome (μg)/microRNA (μg). *; P<0.05.

Cytotoxicity of the various formulations in Hum-63 cells was investigated (Fig .2A). As the results show toxicity was elevated with increasing DOTAP concentration innano-liposomes. These properties have also been approvedwithin 72 hours of treatment. According to these results, F2 formula has a very good positive charge, while it has avery high toxicity effect on the tested cells; so the formulais not satisfactory. In contrast, F6 formula has both positive charge and low toxicity. Therefore, it was selected as the optimal formula. PEG binds to the phospholipid chains and improves liposomal absorption and transportation (19-21). Surface modification of liposomes with PEG also improved biocompatibility and intracellular oligonucleotide stability (21). The length of PEG chain should be optimized to overcome the problems associated with entering as well as escaping the liposomal formulations from endosomal tract, to create a PEG shield. PEGylation process increased the size of nano-liposomal products. Consequently, the presence of PEG in the structure of nano-liposomes was optimal and the PEGylation was kept to 3%.

### Optimization of microRNA loaded into nano-liposomes with agarose electrophoresis gel 

As shown, all of the lipoplex particles with lower than 180/1 µg/µg (liposome/microRNA) concentration had moved along the gel. The concentration of 1 µg microRNA per 180 µg of the loaded liposomes was the highest concentration, remaining in the well and had no movement within the gel (Fig .2B).

### Physical stability of nano-lipoplex containing microRNA 

**Fig.3:**
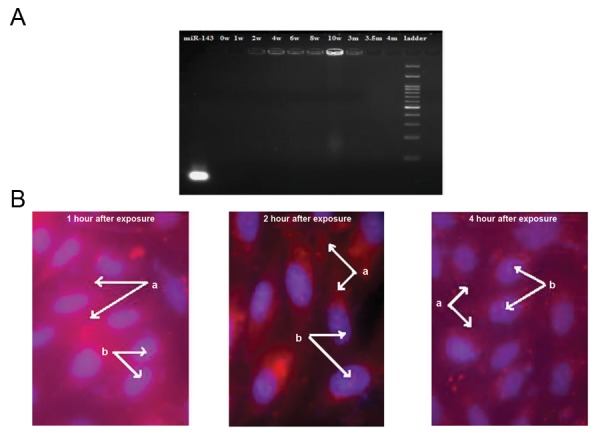
Analysis of the stability test of free microRNA as well as microRNA loaded into lipoplex vesicles and stability of the nano-lipoplex containing microRNA in plasma. A. (Right to left): row 1; free microRNA, row 2; lipoplex -immediately after formation, row 3; lipoplex -after one week, row 4; lipoplex -after two weeks, row 5; lipoplex -after four weeks, row 6; lipoplex -after six weeks, row 7; lipoplex -after eight weeks, row 8; lipoplex -after 10 weeks, row 9; lipoplex -after three months, row 10; lipoplex -after three and half months, row 11; lipoplex -after four months, row 12; Ladder. w; Week. m; Month and B. Nano- lipoplex vesicles were mixed with mouse plasma for one, two and four hours. After this period the SaOs-2 cells were treated with the prepared liposome suspension for one hour at 37°C. *miR-143* was labeled with CY-5 (red) and nucleus was counterstained with DAPI (blue); their merge created a turquoise blue color. a; Liposomal *miR-143* accumulation in the cytoplasm and b; Nuclei stained with DAPI prior to analysis (magnification: ×60).

To determine the stability of nano-lipoplex containing *miR-143*, amount of leakage and release of microRNA from the nano-vesicles, the complex was stored for four months at .4°C temperature. Within a certain time range, the system containing microRNA was sampled and microRNA leakage was monitored by agarose gel electrophoresis. As shown, the system remained stable after four months, and microRNA leakage was not observed by the gel electrophoresis (Fig .3A).

The stability of nano-lipoplex containing microRNA was studied at various time intervals (one, two and four hours) by subjecting it to mouse plasma, to evaluate the ability of formulated liposome in protecting *miR-143* against degradation (Fig .3B). After one, two and four hours contact with plasma, the nano-lipoplex containing microRNA could successfully be absorbed by SaOs-2 cells. Analysis of fluorescence confirms no significant change in the uptake of microRNA with passage of time. 

### Study the effect of lipoplex containing microRNA system

**Fig.4:**
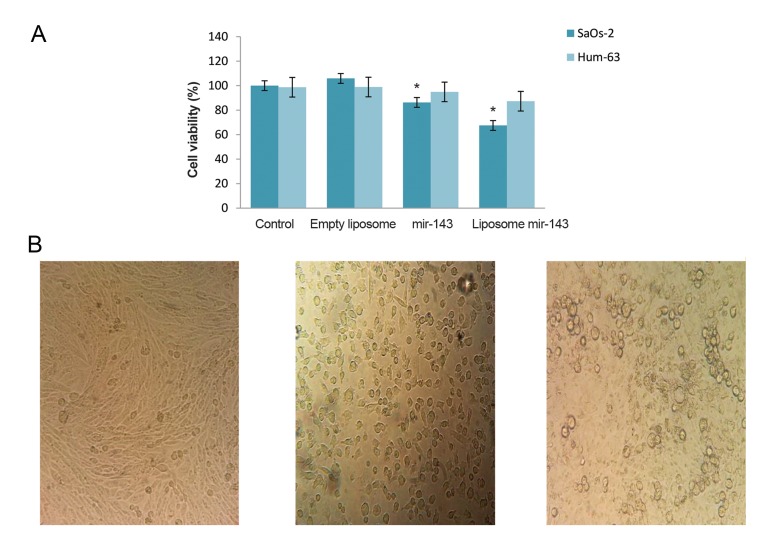
Investigation of lipoplex containing microRNA system effect. **A.** Comparison of free and encapsulated microRNA toxicity after 72 hours, in SaOs-2 and Hum-63 cell lines and **B.** *In vitro* analysis of viability and shape of SaOs-2 cell line. a; With no treatment, b; Treated with free microRNA, and c. Treated with liposomal *miR-143* after 72 hours. *; P< 0.05.

The toxicity rate of Hum-63 and SaOs-2 cell lines at 72 hour is shown in Figure 4A. The empty nano-liposomes, as shown by the previous cell test, are non-toxic. According to the results, microRNA, either free or encapsulated in the liposome, reduced cell growth, while the liposomal formulation had shown more toxicity, especially in SaOs2 cells compared to Hum-63 cell. Viability and shape In vitro of SaOs-2 bone cancer cells treated with free microRNA or encapsulated microRNA after 72 hours is shown in Figure 4B.

### Nano-liposomal microRNA localization assay

**Fig.5:**
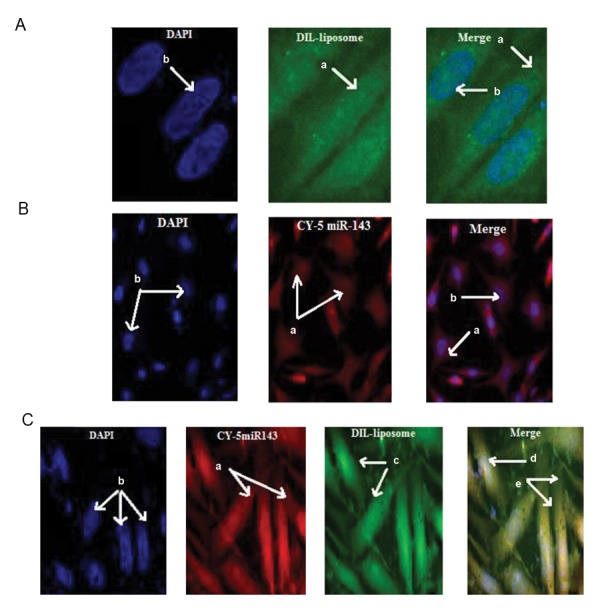
Fluorescence micrographs of cellular uptake, in SaOs-2 cells after three hours treatment. **A.** Empty liposome: a; Accumulation of empty liposomes in the cytoplasm, b; Nuclei stained with DAPI prior to analysis (blue), **B.** Free microRNA: a; Accumulation of the free *miR-143* in the cytoplasm, b; Nuclei stained with DAPI prior to analysis (blue), and C. Liposomal microRNA: a; *miR-143* accumulation in the cytoplasm, b, d; Nuclei stained with DAPI prior to analysis (blue), c; Liposome accumulation in the cytoplasm, and e: liposomal *miR-143* accumulation in the cytoplasm (magnification: ×60).

Cellular uptake of SaOs-2 cells, treated with free microRNA and liposomal *miR-143*, was studied by fluorescence microscopy. As shown in Figure 5, the cells treated with entrapped microRNA showed greater “red color” intensity compared to treated cells with free microRNA. It is well-known that entrapped microRNA (at nano-scale) could penetrate the cells by endocytosis, whereas the free microRNA molecules (at angstrom-scale) were moved by diffusion mechanism. Results showed that SaOs-2 cell line successfully absorbed the entrapped microRNA.

## Discussion

Cancer happens when abnormal cells divide in an uncontrolled way. Osteosarcoma is a cancerous tumor in bone. Osteosarcoma is more common in children and adolescents. Gene therapy is a progressive pathway for transferring genetic material to some cells to correct and manipulate the genome for treatment of various diseases (1, 5, 7, 8, 22). The concept underlying gene therapy is accessible via exogenous DNA, microRNA, short interfering RNA (siRNA) and short hairpin RNA (shRNA). Delivery of free genetic material into the cell generally faces with numerous obstacles. To solve this problem, we tried to design a non-toxic and functional vehicle with high gene loading capacity to provide a specific dose of therapeutic genetic material to target cells. In this regard, optimization and control of the drug agent is important in terms of timing, targeting, dose and maintenance of the therapeutic properties (9, 11, 23).

Several reports indicate *miR-143* is one of the down- regulated microRNAs in different types of cancers, while low levels of *miR-143* have been recognized in many malignant tumors. So, genomic loss of *miR-143* can promote advanced proliferation in cancerous cells (13, 14). In this study, for the first time, we tried to design a cationic liposomal system to transfer *miR-143* into osteosarcoma.

In this study, we evaluated a new formulation of microRNA-cationic transmission system. The liposome structure is based on different values of DPPC, DOTAP, CHOL and DSPE mPEG2000. The new liposomal formulation is stable and prolonged (15). The importance of the compounds used in this formulation has been confirmed in previous reports (16, 17, 24). The particles size in this study was less than 140 nm with and without *miR-143* which is consistent to the reports of Zhang et al. (18) and Nourbakhsh et al. (19).

As a result, PEGylation could improve zeta potential and retained particles at low agglomeration levels. PEGylation also created a shield against cationic scavenging by the macrophage system, thus preventing them to be removed from body’s internal environment (20, 21). It can also partially affect lack of motion of the lipoplex microRNA along the gel. 

The positive charge effect of cationic liposomes inures physical linkage between the gene and liposome. The cationic and neutral properties of liposomes depend on the presence of DOTAP phospholipid. Toxicity of the systems depends on the presence of this phospholipid (17, 18, 25). Cationic lipids are toxic, but in the developed formula in this study, due to its combination with other compounds, such as DPPC, toxicity is diminished (12, 26-29). Similar reports were previously reported by Haghiralsadat et al. (21) and Rehman et al. (30).

Gel electrophoresis confirmed the stability of lipocomplex in environment, by subjecting it to mouse plasma. Additionally, observed fluorescence confirms that no significant change has occurred in the uptake of microRNA with passage of time.

In study the effect of microRNA containing lipoplex system on SaOs-2 cell line, liposomal formulation had shown more toxicity in comparison with free *miR-143*. This is due to the slow releasing behavior of microRNA containing lipoplex, leading to more toxicity for cancer cells as well as a reduction in the use of microRNA and its targeting. Large concentrations of free microRNA small molecules entered into the cells through membrane. However, immediate entry of large volumes of free microRNA into a portion of cell may result in prevention of further import of microRNA from other cellular sites. In the form of nano-lipoplex, microRNA release was limited by slow liberation of nano-liposomal system. As a result, microRNA was gradually introduced to the cell membrane, where it caused uniformity in microRNA absorption into the cell for long-term use (17, 28). This fact help reduce microRNA dosage, due to accumulation at the site. This reduces the amount of drug, needed to treat cancer and increases the therapeutic index along with improvement of cellular toxicity in the SaOS-2 cells. Although application of DOTAP in the formulations leads to a slight reduction of formula biocompatibility, development of these formulations requires preparation of a positive charge formulation for microRNA transport. By optimizing the amount of phospholipid in the formulations, this can be also applied to improve the biocompatibility factor and achieve cationic properties.

The prepared liposomal microRNA formulation could effectively enter the cancerous cells, mostly into the nucleus, whereas free microRNA was predominately distributed in the cytoplasmic region. Accumulation of the *miR-143* in nucleus could induce apoptosis and inhibit DNA replication (29, 30). Concentrating *miR-143* within cancerous cells via carrier system effectively enhanced their anti-cancer activity (31, 32). On the contrary, our results have shown high drug accumulation after three hours treatment. This may be due to smaller size of nanoparticles utilized in our study, leading to increase of diffusion.

## Conclusion

This study suggests a novel and optimized formulation of cationic PEGylated liposomal microRNA for gene delivery. We used small molecule microRNA for evaluating the ability of gene encapsulation, as a delivery system. Cytotoxicity assay showed that toxicity of microRNA, loaded into the liposomes in SaOs-2 cells, was higher than the free form of microRNA, due to the presence of positive surface charge. Cationic liposomes had an ability to interact electrostatically with cell membrane with a negative charge. Hence, these structures could easily pass through cell membrane. It is important to increase microRNA efficiency by improving delivery system design. To solve this problem, we developed and characterized various polypeptide-containing formulations and evaluated them for four months stability parameters, size, zeta potential and gene loading efficiency. Thus, we were able to produce a high-loading microRNA-lipoplex system, not agglomerated, capable of being stored at 4°C for four months without significant leakage of microRNA. Transmission of microRNA into the cell was elevated through the lipoplex system, while it had a good biocompatibility against healthy cells. Consequently, the PEGylated nano-liposomal formulation had a high potential for the systematic migration of microRNA and it could improve the intracellular stability of free microRNA. In general, this study suggested a PEGylated nano-liposomal formulation slowly released, while it had nano-scale size, in the range of 100 nm, in the form of mono-dispersed particles.

## References

[1] Torre LA, Bray F, Siegel RL, Ferlay J, Lortet‐Tieulent J, Jemal A (2015). Global cancer statistics, 2012. CA Cancer J Clin.

[2] Bou Kheir T, Futoma-Kazmierczak E, Jacobsen A, Krogh A, Bardram L, Hother C (2011). miR-449 inhibits cell proliferation and is down-regulated in gastric cancer. Mol Cancer.

[3] Naderinezhad S, Amoabediny G, Haghiralsadat F (2017). Co-delivery of hydrophilic and hydrophobic anticancer drugs using biocompatible pH-sensitive lipid-based nano-carriers for multidrug-resistant cancers. RSC Adv.

[4] Jeon SY, Park JS, Yang HN, Woo DG, Park KH (2012). Co-delivery of SOX9 genes and anti-Cbfa-1 siRNA coated onto PLGA nanoparticles for chondrogenesis of human MSCs. Biomaterials.

[5] Kupcsik L, Stoddart MJ, Li Z, Benneker LM, Alini M (2010). Improving chondrogenesis: potential and limitations of SOX9 gene transfer and mechanical stimulation for cartilage tissue engineering. Tissue Eng Part A.

[6] Santos JL, Pandita D, Rodrigues J, Pêgo AP, Granja PL, Tomás H (2011). Non-viral gene delivery to mesenchymal stem cells: methods, strategies and application in bone tissue engineering and regeneration. Curr Gene Ther.

[7] Oliveira AC, Ferraz MP, Monteiro FJ, Simões S (2009). Cationic liposome- DNA complexes as gene delivery vectors: development and behaviour towards bone-like cells. Acta Biomater.

[8] Tamaki H, Harashima N, Hiraki M, Arichi N, Nishimura N, Shiina H (2014). Bcl-2 family inhibition sensitizes human prostate cancer cells to docetaxel and promotes unexpected apoptosis under caspase-9 inhibition. Oncotarget.

[9] Scholz C, Wagner E (2012). Therapeutic plasmid DNA versus siRNA delivery: common and different tasks for synthetic carriers. J Control Release.

[10] van Kouwenhove M, Kedde M, Agami R (2011). MicroRNA regulation by RNA-binding proteins and its implications for cancer. Nat Rev Cancer.

[11] Soriano A, Jubierre L, Almazán-Moga A, Molist C, Roma J, de Toledo JS (2013). microRNAs as pharmacological targets in cancer. Pharmacol Res.

[12] Gui T, Shen K (2012). miRNA-101: a potential target for tumor therapy. Cancer Epidemiol.

[13] Wang L, Shi ZM, Jiang CF, Liu X, Chen QD, Qian X (2014). MiR-143 acts as a tumor suppressor by targeting N-RAS and enhances temozolomide- induced apoptosis in glioma. Oncotarget.

[14] Wu XL, Cheng B, Li PY, Huang HJ, Zhao Q, Dan ZL (2013). Micro- RNA-143 suppresses gastric cancer cell growth and induces apoptosis by targeting COX-2. World J Gastroenterol.

[15] Soema PC, Willems GJ, Jiskoot W, Amorij JP, Kersten GF (2015). Predicting the influence of liposomal lipid composition on liposome size, zeta potential and liposome-induced dendritic cell maturation using a design of experiments approach. Eur J Pharm Biopharm.

[16] Bao Y, Jin Y, Chivukula P, Zhang J, Liu Y, Liu J (2013). Effect of PEGylation on biodistribution and gene silencing of siRNA/lipid nanoparticle complexes. Pharm Res.

[17] Haghiralsadat F, Amoabediny G, Helder MN, Naderinezhad S, Sheikhha MH, Forouzanfar T (2018). A comprehensive mathematical model of drug release kinetics from nano-liposomes, derived from optimization studies of cationic PEGylated liposomal doxorubicin formulations for drug-gene delivery. Artif Cells Nanomed Biotechnol.

[18] Zhang W, Peng F, Zhou T, Huang Y, Zhang L, Ye P (2015). Targeted delivery of chemically modified anti-miR-221 to hepatocellular carcinoma with negatively charged liposomes. Int J Nanomedicine.

[19] Nourbakhsh M, Behravan J, Lage H, Abnous K, Mosaffa F, Badiee A (2015). Nanolipoparticles-mediated MDR1 siRNA delivery: preparation, characterization and cellular uptake. Nanomed J.

[20] Huang Y, Chen J, Chen X, Gao J, Liang W (2008). PEGylated synthetic surfactant vesicles (Niosomes): novel carriers for oligonucleotides. J Mater Sci Mater Med.

[21] Haghiralsadat F, Amoabediny G, Sheikhha MH, Zandieh‐Doulabi B, Naderinezhad S, Helder MN (2017). New liposomal doxorubicin nanoformulation for osteosarcoma: drug release kinetic study based on thermo and pH sensitivity. Chem Biol Drug Des.

[22] Mitra AK, Agrahari V, Mandal A, Cholkar K, Natarajan C, Shah S (2015). Novel delivery approaches for cancer therapeutics. J Control Release.

[23] Sun NF, Liu ZA, Huang WB, Tian AL, Hu SY (2014). The research of nanoparticles as gene vector for tumor gene therapy. Crit Rev Oncol Hematol.

[24] Saenz del Burgo L, Pedraz JL, Orive G (2014). Advanced nanovehicles for cancer management. Drug Discov Today.

[25] Bose RJ, Arai Y, Ahn JC, Park H, Lee SH (2015). Influence of cationic lipid concentration on properties of lipid-polymer hybrid nanospheres for gene delivery. Int J Nanomedicine.

[26] Cui S, Wang B, Zhao Y, Chen H, Ding H, Zhi D (2014). Transmembrane routes of cationic liposome-mediated gene delivery using human throat epidermis cancer cells. Biotechnol Lett.

[27] Filion MC, Phillips NC (1997). Toxicity and immunomodulatory activity of liposomal vectors formulated with cationic lipids toward immune effector cells. Biochim Biophys Acta.

[28] Tivnan A, Orr WS, Gubala V, Nooney R, Williams DE, McDonagh C (2012). Inhibition of neuroblastoma tumor growth by targeted delivery of microRNA-34a using anti-disialoganglioside GD2 coated nanoparticles. PLoS One.

[29] Haghiralsadat F, Amoabediny G, Naderinezhad S, Forouzanfar T, Helder MN, Zandieh-Doulabi B (2018). Preparation of PEGylated cationic nanoliposome-siRNA complexes for cancer therapy.Artif Cells Nanomed Biotechnol.

[30] Rehman Zu, Zuhorn IS, Hoekstra D (2013). How cationic lipids transfer nucleic acids into cells and across cellular membranes: recent advances. J Control Release.

[31] Khatri N, Baradia D, Vhora I, Rathi M, Misra A (2014). Development and characterization of siRNA lipoplexes: effect of different lipids, in vitro evaluation in cancerous cell lines and in vivo toxicity study. AAPS PharmSciTech.

[32] Kibria G, Hatakeyama H, Sato Y, Harashima H (2016). Anti-tumor effect via passive anti-angiogenesis of PEGylated liposomes encapsulating doxorubicin in drug resistant tumors. Int J Pharm.

